# Supplementary material for: Discovery and application of insertion-deletion (INDEL) polymorphisms for QTL mapping of early life-history traits in Atlantic salmon
Source: BMC Genomics. 2010 Mar 8;11:156. doi: 10.1186/1471-2164-11-156 (PMC2838853; doi:10.1186/1471-2164-11-156)
Supplement: Additional file 2 — Information on developed 76 locus single-run INDEL panel in Atlantic salmon. Information on fluorescence labeling, primer concentrations, PCR pooling and links to alignments, INDEL motifs and GENESCAN (Burge and Karlin 1997) predictions of genes/exons are available in html format. [file 1471-2164-11-156-S2.ZIP › Additionalfile2/snpsummary3355.html]

```
Cluster 697 Contig 2

prev  Summary    Contig List  next
```

Size of Consensus sequence = 2436

Number of sequences = 25

Minimum redundancy = 6

Key

A gi|85161580|gb|DW591281.1|DW591281 smus1-009AB12.y1\_#0537.2 DIAS\_SMUS Salmo salar cDNA 5', mRNA sequence  
B gi|85160292|gb|DW590702.1|DW590702 smus1-004AH10.g1\_#0004.0 DIAS\_SMUS Salmo salar cDNA 5', mRNA sequence  
C gi|45327177|gb|CK897444.1|CK897444 SGP154609 Atlantic salmon Testis cDNA library Salmo salar cDNA clone MG5-0193 5', mRNA sequence  
D gi|117830437|gb|EG903133.1|EG903133 EST\_ssal\_evf\_41721 ssalevf mixed\_tissue Salmo salar cDNA Salmo salar cDNA clone ssal\_evf\_555\_269\_fwd 3', mRNA sequence  
E gi|117477960|gb|EG810179.1|EG810179 EST\_ssal\_evd\_30481 ssalevd thymus Salmo salar cDNA Salmo salar cDNA clone ssal\_evd\_540\_034\_fwd 3', mRNA sequence  
F gi|29314106|gb|CB502880.1|CB502880 ssalmgd501031 gut Salmo salar cDNA, mRNA sequence  
G gi|117463306|gb|EG795525.1|EG795525 EST\_ssal\_evd\_15293 ssalevd thymus Salmo salar cDNA Salmo salar cDNA clone ssal\_evd\_519\_150\_fwd 3', mRNA sequence  
H gi|117463307|gb|EG795526.1|EG795526 EST\_ssal\_evd\_15294 ssalevd thymus Salmo salar cDNA Salmo salar cDNA clone ssal\_evd\_519\_150\_rev 5', mRNA sequence  
I gi|117545811|gb|EG877256.1|EG877256 EST\_ssal\_eve\_20891 ssaleve thyroid Salmo salar cDNA Salmo salar cDNA clone ssal\_eve\_528\_153\_fwd 3', mRNA sequence  
J gi|29310665|gb|CB499439.1|CB499439 ssalkhc009064 kidney Salmo salar cDNA, mRNA sequence  
K gi|117830436|gb|EG903132.1|EG903132 EST\_ssal\_evf\_41720 ssalevf mixed\_tissue Salmo salar cDNA Salmo salar cDNA clone ssal\_evf\_555\_269\_rev 5', mRNA sequence  
L gi|29318181|gb|CB506535.1|CB506535 ssalmna006006 muscle\_skin Salmo salar cDNA, mRNA sequence  
M gi|117545810|gb|EG877255.1|EG877255 EST\_ssal\_eve\_20890 ssaleve thyroid Salmo salar cDNA Salmo salar cDNA clone ssal\_eve\_528\_153\_rev 5', mRNA sequence  
N gi|117462671|gb|EG794890.1|EG794890 EST\_ssal\_evd\_14720 ssalevd thymus Salmo salar cDNA Salmo salar cDNA clone ssal\_evd\_518\_226\_rev 5', mRNA sequence  
O gi|117477959|gb|EG810178.1|EG810178 EST\_ssal\_evd\_30480 ssalevd thymus Salmo salar cDNA Salmo salar cDNA clone ssal\_evd\_540\_034\_rev 5', mRNA sequence  
P gi|117462672|gb|EG794891.1|EG794891 EST\_ssal\_evd\_14721 ssalevd thymus Salmo salar cDNA Salmo salar cDNA clone ssal\_evd\_518\_226\_fwd 3', mRNA sequence  
Q gi|117828834|gb|EG901530.1|EG901530 EST\_ssal\_evf\_40278 ssalevf mixed\_tissue Salmo salar cDNA Salmo salar cDNA clone ssal\_evf\_553\_297\_rev 5', mRNA sequence  
R gi|117828835|gb|EG901531.1|EG901531 EST\_ssal\_evf\_40279 ssalevf mixed\_tissue Salmo salar cDNA Salmo salar cDNA clone ssal\_evf\_553\_297\_fwd 3', mRNA sequence  
S gi|29313406|gb|CB502180.1|CB502180 ssalmge506257 gut Salmo salar cDNA, mRNA sequence  
T gi|45311423|gb|CK881792.1|CK881792 SGP142143 Atlantic salmon Head kidney cDNA library Salmo salar cDNA clone FN4-1309 5', mRNA sequence  
U gi|89861166|gb|DY717289.1|DY717289 EST\_ssal\_rgb2\_73028 ssalrgb2 mixed\_tissue Salmo salar cDNA Salmo salar cDNA clone ssal\_rgb2\_618\_100\_rev 5', mRNA sequence  
V gi|85053053|gb|DW581231.1|DW581231 EST\_ssal\_rgb2\_45650 rgb2 Salmo salar cDNA clone ssal\_rgb2\_573\_329\_rev 5', mRNA sequence  
W gi|24390156|gb|CA059913.1|CA059913 ssalrgb505227 mixed\_tissue Salmo salar cDNA, mRNA sequence  
X gi|85053052|gb|DW581230.1|DW581230 EST\_ssal\_rgb2\_45649 rgb2 Salmo salar cDNA clone ssal\_rgb2\_573\_329\_fwd 3', mRNA sequence  
Y gi|24354304|gb|CA048134.1|CA048134 ssalkhc002043 kidney Salmo salar cDNA, mRNA sequence

2 SNPs detected

A B C D E F G H I J K L M N O P Q R S T U V W X Y  cosegregation weighted

683 . . . - - C C C C - - - C - . - C C C . . . . . .   2/2 60.00
684 . . . - - T T T T - - - T - . - T T T . . . . . .   2/2 60.00
